# The revision hip arthroscopy complex: capsular deficiency, labral deficiency, femoral over-resection and adhesions can result in good survivorship with revision hip arthroscopy

**DOI:** 10.1093/jhps/hnad026

**Published:** 2023-09-09

**Authors:** Heath P Melugin, Spencer M Comfort, Trevor S Shelton, Hannah K Day, Joseph J Ruzbarsky, Grant J Dornan, Marc J Philippon

**Affiliations:** Center for Outcomes-Based Orthopaedic Research, Steadman Philippon Research Institute, 181 W Meadow Dr, Ste 1000, Vail, CO 81657, USA; Steadman Clinic and United States Coalition for the Prevention of Illness and Injury in Sport, 181 W Meadow Dr, Ste 400, Vail, CO 81657, USA; Center for Outcomes-Based Orthopaedic Research, Steadman Philippon Research Institute, 181 W Meadow Dr, Ste 1000, Vail, CO 81657, USA; Steadman Clinic and United States Coalition for the Prevention of Illness and Injury in Sport, 181 W Meadow Dr, Ste 400, Vail, CO 81657, USA; Center for Outcomes-Based Orthopaedic Research, Steadman Philippon Research Institute, 181 W Meadow Dr, Ste 1000, Vail, CO 81657, USA; Center for Outcomes-Based Orthopaedic Research, Steadman Philippon Research Institute, 181 W Meadow Dr, Ste 1000, Vail, CO 81657, USA; Steadman Clinic and United States Coalition for the Prevention of Illness and Injury in Sport, 181 W Meadow Dr, Ste 400, Vail, CO 81657, USA; Center for Outcomes-Based Orthopaedic Research, Steadman Philippon Research Institute, 181 W Meadow Dr, Ste 1000, Vail, CO 81657, USA; Center for Outcomes-Based Orthopaedic Research, Steadman Philippon Research Institute, 181 W Meadow Dr, Ste 1000, Vail, CO 81657, USA; Steadman Clinic and United States Coalition for the Prevention of Illness and Injury in Sport, 181 W Meadow Dr, Ste 400, Vail, CO 81657, USA

## Abstract

To evaluate the patient-reported outcomes (PROs) and survivorship of combined arthroscopic hip labral reconstruction/augmentation, capsular reconstruction, femoral neck remplissage and lysis of adhesions. Patients ≥18 years old who underwent this combination of procedures during revision hip arthroscopy and were eligible for minimum 2-year follow-up were identified. PRO scores including Hip Outcome Score (HOS)-Activities of Daily Living scale, HOS-Sports scale, modified Harris Hip Score, Short Form 12, and Western Ontario & McMaster Universities Osteoarthritis Index, patient satisfaction and failure rates were analyzed. Seven patients (5 females and 2 males) with average age of 45.0 ± 5.2 (range: 40–54 years) met inclusion criteria. Patients had a median of 1 (range: 1–3) prior hip surgery at an outside institution. All patients had previously undergone femoral osteoplasty, and 85% (6/7) of patients had a labral repair performed. Four patients had no capsule closure performed in their prior procedures. Six patients were available for minimum 2-year follow-up. Two patients converted to total hip arthroplasty: one patient with four prior hip arthroscopies and the other had advanced osteoarthritis with outerbridge grade 3/4 defects requiring microfracture. Mean patient satisfaction was 7 (range: 2–9). At mean follow-up of 3 years, most patients who underwent the combination of labral reconstruction, capsular reconstruction, femoral neck remplissage and lysis of adhesions during revision hip arthroscopy demonstrated improved PROs. This salvage procedure has the potential to restore hip function in patients who have failed an initial hip arthroscopy procedure. In patients with these pathologies present and concomitant joint space narrowing, a total hip arthroplasty may be a more appropriate salvage option.

## INTRODUCTION

With an increasing incidence of primary hip arthroscopy, the incidence of revision hip arthroscopy has increased as well [1]. While residual femoroacetabular impingement (FAI) due to cam under-resection is the most common indication for revision hip arthroscopy, other causes are common [2, 3]. Other indications for revision hip arthroscopy include persistent hip pain due to residual FAI with a recurrent labral tear and microinstability relating to labral deficiency, capsular deficiency, adhesion formation and/or cam over-resection [2].

Revision hip arthroscopy, like most other revision procedures, does not have the same degree of success as its primary counterparts [4–6]. Newman et al. [4] performed a matched-cohort study comparing the results of primary and revision hip arthroscopy patients. Both groups had improved outcomes, but those undergoing revision surgery had lower patient-reported outcome (PRO) scores. Advanced procedures, including labral reconstruction, capsular reconstruction and femoral remplissage have been developed to address these issues found in revision hip arthroscopy [7–9]. These procedures can be complicated and require significant surgeon expertise, but have demonstrated great functional outcomes and survivorship [10–12]. Although rare, the hip arthroscopist should be prepared to encounter several of these issues in the same patient. Unfortunately, there is a paucity of evidence regarding survivorship and PROs of these combined procedures which can help in surgical decision making and patient counseling.

The purpose of this case series is to evaluate the PROs and survivorship of arthroscopic hip labral reconstruction/augmentation, capsular reconstruction, femoral neck remplissage and lysis of adhesions for treatment of the terrible combination of labral deficiency, capsular deficiency, cam over-resection, and adhesions. It is hypothesized that patients who undergo this combination of procedures will have high arthroplasty-free survivorship and improvement in PROs at a minimum of 2-year follow-up.

## METHODS

### Study design

This study was approved by the institutional review board at the authors’ institution (Vail Health). Cases of labral reconstruction or augmentation, capsular reconstruction, remplissage of the femoral head/neck and lysis of adhesions during revision hip arthroscopy with the senior surgeon (M.J.P.) between January 2013 and February 2020 were identified from a prospectively collected database. Individuals were included if they were at least 18 years old at the time of surgery and were eligible for minimum 2-year follow-up. Patients were excluded if all four of these procedures were not performed during the same surgery. The outcomes of these procedures have been previously reported on individually by this study group, so there is overlap between the present study’s patient population and those reported on in prior studies given the present study’s inclusion criteria and study dates [11–14].

Demographic data, symptom history, physical examination findings, radiographic findings, concomitant procedures (including microfracture) and surgical details were collected. Survivorship prior to subsequent hip arthroscopy and/or arthroplasty was collected. PROs were collected pre- and post-operatively and included modified Harris Hip Score (mHHS), Hip Outcome Score (HOS) Activities of Daily Living (ADL) and Sport subscales, Vail Hip Score, Visual Analog Score (VAS), Western Ontario and McMaster Universities Osteoarthritic Index (WOMAC) and the 12-item Short Form Survey (SF-12).

### Clinical evaluation

Patients with continued pain, dysfunction, and/or instability following previous hip arthroscopy underwent clinical evaluation with thorough history, physical examination and diagnostic testing. Operative reports were collected from outside institutions to determine previous procedures performed on the hip. Physical examination findings indicative of intraarticular pathology included a positive flexion adduction internal rotation (FADIR) test, flexion abduction external rotation (FABER) distance test and dial test [15]. Positive FADIR test is thought to be due to the severe disruption of the fluid seal and lack of hip congruence, which results in translational movement of the femoral head when the hip is brought into extreme flexion and adduction that can cause gross instability and contra-coup pain [16]. Positive FABER distance test is indicative of symptomatic femoroacetabular impingement [17]. Positive dial test indicates increased capsular laxity or deficiency [18].

Radiographic examination included anteroposterior (AP) pelvis, false profile and a 45° Dunn views. These radiographs allowed for measurement of lateral center-edge angle (LCEA), anterior center-edge angle (ACEA), Sharp’s angle, Tönnis angle and alpha angle. LCEA and ACEA angles less than 20 degrees were indicated for combined periacetabular osteotomy and hip arthroscopy with the decision to proceed shared between the surgeon and patient. Magnetic resonance imaging (MRI) was used to assess for labral bulk and integrity, articular cartilage integrity, structural abnormalities of the capsule, presence of cam over-resection and other soft tissue pathology. Femoral neck over-resection was determined on AP pelvis and 45°Dunn views as well as MRI as the greatest dimension of over-resection was not always readily apparent on plain radiographs. Presence of over-resection was confirmed intraoperatively using a dynamic examination to determine the presence or disruption of the suction seal. Physical examination was performed by the senior author while radiographic measurements were performed by orthopedic sports medicine fellows. Patients with intraarticular pain refractory to physical therapy and other conservative treatments and evidence of labral and capsular insufficiency with femoral neck over-resection were indicated for surgery.

### Surgical technique

Patients received the four-component treatment if they were found to have (i) deficiency of the labrum with a labral width less than 6 mm and/or of poor tissue integrity not amenable to stable repair (this includes cases of ossified labrum); (ii) prior cam over-resection as evidenced by a femoral neck defect appreciated intraoperatively with significant impairment of the fluid seal during intraoperative dynamic hip examination in hip flexion and/or abduction; (iii) capsular defect or deficiency lacking adequate tissue volume and/or quality for repair and closure; and (iv) presence of capsulolabral adhesions. The labrum reconstruction/augmentation was performed with iliotibial band (ITB) allograft or autograft, and both the remplissage and capsular reconstruction were performed with ITB allograft.

**Fig. 1.**
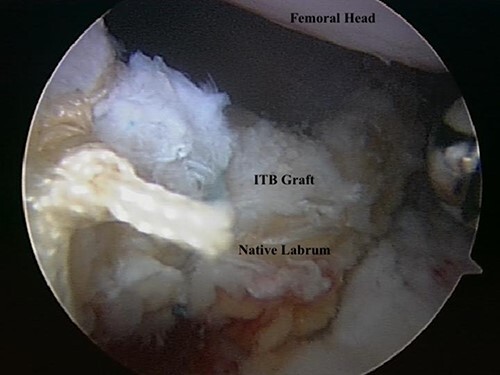
Arthroscopic photo demonstrating a completed labral augmentation with iliotibial band autograft.

The techniques for labral reconstruction/augmentation, capsular reconstruction and remplissage have been previously described [7–9]. Briefly, patients were placed in a modified supine position (10° of flexion, 15° of internal rotation, 10° of lateral tilt and neutral abduction). Hip arthroscopy was performed using anterolateral and mid-anterior portals and a 2.5-cm inter-portal capsulotomy. A diagnostic arthroscopy was performed including a dynamic exam to evaluate the labral seal mechanism. The size of the labral defect was measured with an arthroscopic probe to determine the size of the graft. Once harvested and prepared, the labral graft was shuttled into the joint and first fixed at the most medial aspect of the labral defect followed by fixation of the lateral side of the graft to the acetabulum, using additional suture anchors every 7–10 mm to fixate the labral graft (Fig. 1). A dynamic hip examination was then performed to assess the region of suspected over-resection.

**Fig. 2.**
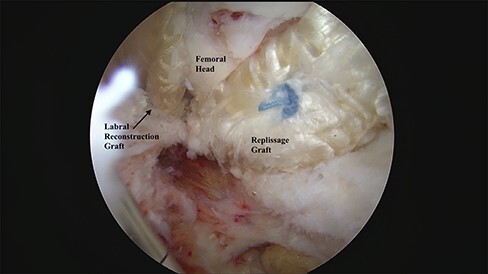
Arthroscopic photo of the femoral neck after remplissage with iliotibial band allograft.

The region of over-resection was gently decorticated to a bleeding bony base to facilitate healing without removing additional bone, and the dimensions of the defect were measured. The ITB allograft was then prepared and contoured to fit the defect as described previously [8]. The remplissage graft was shuttled into the joint and secured to an all-suture knotted anchor placed in the center of the defect of the femoral neck. Additional all-suture anchors were placed every 5–7 mm in a two-dimensional meshwork fashion to secure the graft (Fig. 2). Another dynamic examination was then performed to confirm appropriate filling of the defect and re-establishment of the suction seal.

**Fig. 3.**
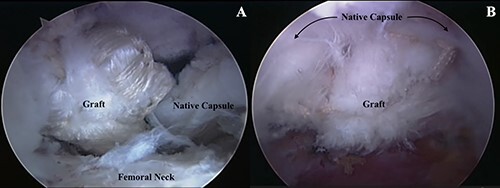
Arthroscopic photos of the capsular reconstruction iliotibial allograft after proximal fixation to the acetabular rim with suture anchors (A) and after securing the graft to the native capsule (B).

Attention was then turned to the capsule. The quality and quantity of capsular tissue remaining was evaluated. If the remaining tissue was not adequate for repair then the decision was made to proceed with capsular reconstruction. The size of the capsular defect was measured and the ITB allograft was prepared as previously described [9]. The graft was inserted and fixed on the acetabular rim near the 3-o’clock position and the graft shuttled into place with a tension slide technique. The proximal lateral extent of the capsular graft was then fixed in place with an additional suture anchor, often near the 12-o’clock position. A final anchor was placed between the previously fixed ends. The free sides of the graft were sutured to the native capsular tissue using the SutureLasso device (Arthrex, Naples, FL) and the Arthro-Pierce device (Smith & Nephew, Andover, MA) without any distal bony fixation (Fig. 3). Finally, a last dynamic examination was then performed to ensure proper fixation and repair of the capsule defect.

### Rehabilitation

All patients followed a similar rehabilitation protocol with emphasis on early passive hip mobilization and circumduction for the prevention of adhesions [19]. Patients were placed in a continuous passive motion machine immediately after surgery and used it daily for 4 weeks to reduce the incidence of adhesions. A hip brace was worn for 17 days to prevent excessive hyperextension and abduction. Anti-rotational bolsters were used at night for 3 weeks. During the first 2 weeks, range of motion restrictions included abduction from 0° to 45° and flexion to 90° without hyperextension. Patients were limited to flat-foot weight-bearing to less than 20 pounds for 3–4 weeks (unless microfracture is performed then at least 6 weeks). Return to full function and sports was permitted when the patient completed all stages of the rehabilitation protocol and was able to pass the Hip Sports Test [19].

### Statistical analysis

Mean and standard deviations were calculated with standard formulas. Statistical analysis was performed using SPSS software (version 28, SPSS Inc., Chicago, IL).

## RESULTS

Seven patients met inclusion criteria. Patient demographics are listed in Table I. There were five females and two males with mean age of 45 ± 5 years (range: 39–54). Patients had a median of 1 (range: 1–3) prior hip surgeries, all performed at outside institutions, before undergoing hip arthroscopy with the senior surgeon at mean 2.9 ± 2.4 years (range: 1.3–8.2) following their previous surgery. Operative reports were available for all previous surgeries. Procedures performed are described in Table I. All patients had undergone femoral osteoplasty for treatment of a cam lesion. One patient had labral debridement. Five patients had a labral repair with suture anchors. One patient had a primary labral reconstruction performed. Seven of 10 (70%) prior hip arthroscopies (accounting for multiple surgeries in some patients) did not include capsule closure. Four patients (57%) had no type of capsulotomy repair in the most recent prior surgery.

**Table I.** Patient characteristics and surgical history of ipsilateral hip prior to hip arthroscopy with combination of procedures

Patient	Sex	Age	Years from previous surgery to index surgery	Number of prior ipsilateral hip surgeries	Description of procedures performed in prior surgeries
**1**	F	41	1.8	1	Labral repair, acetabular microfracture, femoral osteoplasty, no capsular closure
**2**	F	46	5.0	1	Labral repair, rim trimming, acetabular chondroplasty, partial synovectomy, femoral osteoplasty, psoas release, loose body removal, capsular plication
**3**	F	40	2.0	2	1st: isolated PAO 2nd: labral repair, rim trimming, femoral head chondroplasty, femoral osteoplasty, capsular closure
**4**	M	49	8.2	1	Labral reconstruction, rim trimming, femoral osteoplasty, acetabular chondroplasty, synovectomy, no capsule closure
**5**	M	44	1.4	2	1st: labral repair, rim trimming, synovectomy, acetabular chondroplasty, anterior inferior iliac spine subspinous decompression, femoral osteoplasty, ligamentum teres debridement, no capsule closure 2nd: synovectomy, lysis of adhesions, no capsule closure
**6**	F	39	1.5	3	1st: labral repair, no capsule closure 2nd: labral debridement, no capsule closure 3rd: labral repair, lysis of adhesions, femoral osteoplasty, capsular closure
**7**	F	54	1.3	1	Labral debridement, femoral osteoplasty, loose body removal, lysis of adhesions, femoral head chondroplasty, no capsule closure

PAO = periacetabular osteotomy.

Table II displays the pre-operative radiographic measurements of the patients. Pre-operatively, two patients had Tönnis grade 0, four patients had grade 1 and one patient had grade 2. The majority of patients had radiographic joint space >2 mm (6/7) with the one patient having a minimum radiographic joint space of 1.2 mm with all other measurements in the same patient being above 2 mm. Average alpha angle on pre-operative radiographs was 52± degrees (range: 31–74). The areas of over-resection were focal in nature and there were regions where there was residual impingement on some of the patients; therefore, patients presented with simultaneous over- and under-resection. Intraoperative findings and procedures performed in addition to labral reconstruction, femoral neck remplissage and capsular reconstruction can be found in Table III. The size of capsular, labral and femoral neck defects measured as well as graft inserted are listed for each patient in Table III.

**Table II.** Pre-operative radiographic measurements for patients undergoing combination of procedures during hip arthroscopy

Patient	Alpha angle, degrees	CEA, degrees	Sharp angle, degrees	Tönnis angle, degrees	Tönnis grade	Minimum joint space, mm
**1**	59	25	42	9.1	0	3.9
**2**	47	28	43	2.3	1	3.7
**3**	57	47	42	3.1	1	3.0
**4**	62	22	39	7.7	1	3.0
**5**	35	19	45	13	0	3.6
**6**	31	39	31	0.1	1	2.1
**7**	74	36	34	2.5	2	1.2

CEA = center-edge angle.

**Table III.** Graft sizes and concomitant procedures performed during hip arthroscopy with combination of procedures

Patient	Size of graft (mm) placed for capsular, labral and femoral neck defects during revision surgery	Concomitant procedures
**1**	C: 50 × 30 L: 40 × 9 FN: 7 × 30 × 4	LOA, rim trimming, subspinal decompression, focal femoral osteoplasty, acetabular chondroplasty, synovectomy, ligamentum teres debridement
**2**	C: 20 × 20 L: 40 × 6 FN: 20 × 20 × 7	LOA, rim trimming, subspinal decompression, focal femoral osteoplasty, acetabular chondroplasty, synovectomy, ligamentum teres debridement
**3**	C: 20 × 20 L: 55 × 8 FN: 20 × 10 × 4	LOA, rim trimming, subspinal decompression, focal femoral osteoplasty, acetabular chondroplasty, femoral head chondroplasty, synovectomy, ligamentum teres debridement
**4**	C: 40 × 30 L: 15 × 3 FN: 20 × 10 × 2	LOA, rim trimming, subspinal decompression, focal femoral osteoplasty, acetabular chondroplasty, femoral head chondroplasty, synovectomy, ligamentum teres debridement
**5**	C: 22 × 22 L: 45 × 8 FN: 22 × 10 × 3.5	LOA, rim trimming, subspinal decompression, focal femoral osteoplasty, femoral head chondroplasty, synovectomy, ligamentum teres debridement
**6**	C: 60 × 30 L: 50 × 7 FN: 20 × 10 × 4	LOA, rim trimming, subspinal decompression, focal femoral osteoplasty, acetabular chondroplasty, synovectomy, ligamentum teres debridement, trochanteric bursectomy, iliotibial band release
**7**	C: 30 × 38 L: 40 × 7 FN: 20 × 10 × 2	LOA, rim trimming, subspinal decompression, focal femoral head chondroplasty, acetabular chondroplasty, synovectomy, ligamentum teres debridement, trochanteric bursectomy, removal of loose bodies

C = capsular defect; FN = femoral neck defect; L = labral defect; LOA = lysis of adhesions.

Minimum 2-year follow-up was obtained for six patients (85%). Two patients had converted to total hip arthroplasty (Patients 6 and 7). One patient converted at 1.8 years with four prior hip arthroscopies. The other patient converted at 4.7 years, having a minimum joint space of 1.2 mm and bipolar Outerbridge grade 3/4 defects requiring acetabular microfracture at the time of his revision. For the remaining four patients (Patients 1–4), average follow-up time was 3.2 ± years. Pre-operative and post-operative outcome scores are listed in Table IV.

**Table IV.** Patient-reported outcomes after revision hip arthroscopy with combination of procedures

	HOS-ADL	HOS-Sport	mHHS	WOMAC	SF-12 PCS	SF-12 MCS	Satisfaction
Patient 1 Pre-op score Post-op score	70 85	47 47	57 82	24 13	44.5 52.0	60.0 57.8	8
Patient 2 Pre-op score Post-op score	65 57	33 55	37 56	46 45	40.2 39.9	36.3 56.5	2
Patient 3 Pre-op score Post-op score	65 79	44 61	55 72	35 31	45.5 54.6	60.3 51.9	7
Patient 4 Pre-op score Post-op score	75 92	61 83	79 74	17 1	51.4 56.9	56.6 55.6	9

HOS-ADL= Hip Outcome Score Activities of Daily Living; HOL-Sport= Hip Outcome Score Sport Score; mHHS= modified Harris Hip Score; SF-12 MCS = 12-Item Short Form Mental Component Score; SF-12 PCS= 12-Item Short Form Physical Component Score; WOMAC = Western Ontario and McMaster Universities Osteoarthritis Index.

## DISCUSSION

The most important finding of this study was that patients undergoing revision hip arthroscopy in the salvage situation of labral and capsular deficiency with cam over-resection and adhesions can obtain successful outcomes with good survivorship. This study found improved PROs in the majority of patients at mean follow-up of 3.2 years. As for survivorship, two patients converted to total hip arthroplasty, one after four prior hip arthroscopies and the other had evidence of severe bipolar chondral defects at time of the index surgery. While the combination of labral reconstruction, capsular reconstruction, femoral neck remplissage and lysis of adhesions can restore hip stability and fluid seal mechanism and improve symptoms after previous failed hip arthroscopies, it is a salvage procedure and patients should be counseled appropriately.

The terrible combination of labral deficiency, capsular deficiency, cam over-resection and adhesions is extremely detrimental to hip function and must be addressed surgically when non-operative treatments fail. Each pathology in isolation can contribute to microinstability and hip fluid seal dysfunction, but the combination likely acts synergistically and leads to significant hip dysfunction and pain [20, 21]. It is important to recognize and address each of these deficiencies at the time of revision surgery in order to best optimize outcomes. The mean age of the patient cohort in this study was middle-aged, 45 years and the majority of patients were found to have only mild osteoarthritis (Tönnis grade of 0, preserved joint space (>2 mm) and minimal severe chondral lesions), which is why the decision was made to proceed with revision hip arthroscopy rather than arthroplasty. Labral reconstruction, capsular reconstruction and femoral neck remplissage have all demonstrated improved PROs individually in previous studies but have yet to be evaluated as concomitant procedures [11–14]. The current study’s results demonstrate these procedures can successfully relieve symptoms and restore function in the hip and should be considered as a salvage, joint preserving procedure for individuals who have minimal osteoarthritis. For patients with concomitant joint space narrowing, a total hip arthroplasty may be a more appropriate salvage option.

Labral deficiency in the revision setting can be related to previous labral debridement, formation of capsulolabral adhesions, residual bony impingement, re-injury and/or degeneration [22]. Decreased labral volume and poor tissue quality, specifically a labral height of <6 mm, has been significantly associated with decreased distance to suction seal rupture and decreased peak negative pressure in biomechanical investigation [23]. Labral reconstruction and augmentation have demonstrated improved pain and function in the revision setting. At 10-year minimum follow-up after labral reconstruction with ITB autograft, Philippon et al. found the mean survival time to be similar between patients who underwent revision surgery versus primary surgery (10.9 versus 9.1 years, *P* = 0.43); however, the survival rate was 90% for the patients with >2 mm of joint space [13]. A recent study by Soares et al. demonstrated improved PROs and high patient satisfaction following labral augmentation during revision hip arthroscopy in 77 patients with a survivorship of 93% at 5 years [24]. With appropriate patient selection, labral augmentation and reconstruction for treatment of labral deficiency can provide symptom relief and improved functional outcomes.

Cam under-resection is the most common indication for revision hip arthroscopy, but cam over-resection is a rare indication [25]. While cam under-resection can lead to continued impingement and chondrolabral damage, cam over-resection can prevent formation of the suction seal and contribute to microinstability [20, 21, 26]. While techniques using radiographic visualization and/or anatomic landmarks have been developed to help guide appropriate cam resection, an intraoperative dynamic hip examination should be performed to verify that bony impingement has been successfully removed and the suction seal has been restored [9, 27–30]. In the unfortunate instances when cam over-resection is identified, remplissage with an ITB allograft can be used to fill the bony defect to reestablish anatomic contact between the labrum and femoral head–neck junction and restore the suction seal, allowing for improved hip stability [8]. Arner et al. reported on 13 patients who had undergone arthroscopic hip remplissage at mean 3.1-year follow-up and found one patient had converted to Total Hip Arthroplasty (THA) and the other patients had improved PROs with a minimal clinically important difference (MCID) of 83% for mHHS [11].

The capsule plays an important role in hip biomechanics, acting as the primary stabilizer during rotation and preventing over-translation of the femur [31]. While several studies have demonstrated biomechanical advantage and superior patient outcomes with capsular closure following hip arthroscopy, it is not routinely performed in all practices [32–35]. Seven of the nine (78%) prior arthroscopy procedures of the 18 patients in the current study had no capsular closure performed. Such defects, in addition to those as result of capsulectomy, post-operative re-rupture or failure of healing can lead to symptomatic microinstability [36]. The presence of capsulolabral adhesions is another source of microinstability as the capsule can become tethered to the labrum leading to eversion with disruption of the suction seal. Measures to prevent capsular deficiency include proper capsulotomy closure, post-operative circumduction exercises and more recently systemic therapeutics [37, 38]. In cases of capsular deficiency, capsular reconstruction is a surgical option that has led to improved patient outcomes [9]. At mean follow-up of 25 months, Fagotti et al. found patients treated with an ITB autograft had significantly higher post-operative PROs and greater percentage of patients reached MCID for HOS scales and WOMAC compared with those treated with a dermal allograft for capsular reconstruction [14]. Recently, Ruzbarsky et al. demonstrated a cohort of 39 patients with median 2 (range: 0–3) prior hip arthroscopy surgeries had a THA-free survival rate of 86% at 3 years following capsular reconstruction with 90% of patients achieving MCID for HOS-ADL and HOS-Sport at mean follow-up of 4.3 years [12].

Finally, it is important to recognize that patients presenting with the combination of these pathologic findings should be counseled on the alternative of THA, especially those in their 40s and older. In the current study, two patients, age 39 and 54 with minimum joint space of 2.1 and 1.2 mm, respectively, at time of surgery converted to THA. Studies have demonstrated joint space, presence of severe chondral defects and age to be important predictors of failure [39, 40]. Patients at risk of early failure of hip arthroscopy should be educated on the success rates of both procedures, the expected recoveries, as well as the rehabilitation.

### Limitations

This study is not without limitations. First and most importantly, the sample size of the study is small with limited patients eligible for 2-year minimum follow-up. However, it is important to present these findings for surgeons performing hip arthroscopy to bring awareness to this issue and strategize ways to prevent future cases. Second, the patient population represents a heterogeneous group with complex hip pathologies with varying severity of osteoarthritis and prior procedures performed by different surgeons. While operative reports from outside institutions were available, it is difficult to capture all procedures previously performed and the technique used. Furthermore, there was a high proportion of concomitant procedures performed including all patients undergoing lysis of adhesions. These concomitant procedures detract from the strength of conclusions but speak to the high degrees of pathologies encountered in the multiply revised hip scope. Finally, the retrospective nature and lack of a control group are obvious limitations of this small series. Therefore, it may be difficulty to extrapolate the current study results to a larger population.

## CONCLUSION

At mean follow-up of 3 years, most patients who underwent the combination of labral reconstruction, capsular reconstruction, femoral neck remplissage and lysis of adhesions during revision hip arthroscopy demonstrated improved PROs. This salvage procedure has the potential to restore hip function in patients who have failed an initial hip arthroscopy procedure. In patients with these pathologies present and concomitant joint space narrowing, a total hip arthroplasty may be a more appropriate salvage option.

## DATA AVAIILABILITY

The data used to support the findings of this study are included within the article.
